# An Observational Study on the Diagnosis and Antibiotics Prescription in Cats with Lower Urinary Tract Disease by Veterinarians in Italy

**DOI:** 10.3390/vetsci12040313

**Published:** 2025-03-30

**Authors:** Isabella Tirelli, Francesca Fidanzio, Simone Bertini, Serena Crosara, Luigi Intorre, Ilaria Lippi, Veronica Marchetti, Andrea Corsini

**Affiliations:** 1 Department of Veterinary Sciences, University of Parma, 43126 Parma, Italy; isabella.tirelli@unipr.it (I.T.); simone.bertini@unipr.it (S.B.); serena.crosara@unipr.it (S.C.); andrea.corsini@unipr.it (A.C.); 2 Department of Veterinary Medical Sciences, University of Pisa, 56122 Pisa, Italy; luigi.intorre@unipi.it (L.I.); ilaria.lippi@unipi.it (I.L.); veronica.marchetti@unipi.it (V.M.)

**Keywords:** feline lower urinary tract diseases, urinary tract infections, urinalysis, ISCAID, antibiotic, anti-inflammatory, quinolones

## Abstract

Feline lower urinary tract disease (FLUTD) is a frequent diagnosis in cats, with bacterial causes constituting a minor percentage of cases. This observational study aimed to assess the diagnostic and therapeutic approaches to FLUTD among Italian veterinarians and explore variations in different workplaces. Data were collected via an anonymous survey completed by 317 veterinarians from small clinics (SC), large clinics (LC), and veterinary hospitals (VH). Results revealed that approximately half of respondents relied on clinical signs and urinalysis for diagnosis, while fewer incorporated abdominal ultrasound. Urine culture and sensitivity (UCS) testing was infrequently performed, primarily due to financial and logistical constraints, with higher compliance in VH practitioners. Antimicrobials were widely employed, with enhanced penicillins being the most prescribed class. The frequent empirical use of quinolones raises concern regarding gaps in antimicrobial stewardship. Despite some adherence to the ISCAID guidelines, discrepancies in diagnostic rigor, antimicrobial selection, and treatment duration were observed. Larger facilities demonstrated greater alignment with evidence-based practices, emphasizing the need for widespread education on appropriate antimicrobial use. This study underscores the importance of integrating guideline-based diagnostics and stewardship in managing feline urinary tract diseases.

## Simple Summary

This study investigated the diagnostic and therapeutic approaches to feline lower urinary tract disease among Italian veterinarians, emphasizing variations across small clinics, large clinics, and veterinary hospitals. Data were collected through an anonymous questionnaire completed by 317 veterinarians. The primary diagnostic criteria chosen by responders included clinical signs and urinalysis, while urine culture and sensitivity were infrequently performed. Despite the low reported percentage of bacterial cystitis in cats with lower urinary tract signs, antimicrobials were widely prescribed. Deviations from established guidelines were identified, underscoring the necessity for enhanced education on diagnostic procedures, antimicrobial selection, and treatment duration to promote effective antimicrobial stewardship.

## 1. Introduction

Feline lower urinary tract disease (FLUTD) is among the most common diagnosis in cats [1]. Clinical signs include stranguria, pollakiuria, dysuria, and hematuria, with 20–55% of male cats at risk of life-threatening urinary obstruction [2,3,4,5]. These manifestations are collectively termed lower urinary tract signs (LUTS). FLUTD commonly arises from feline idiopathic cystitis (FIC), uroliths, urinary tract infections (UTIs), and anatomical or neurological abnormalities [6]. FIC is the prevalent cause, reported in 55–67% of cases [7], whereas UTIs traditionally account for <2% in young cats [2,5,8,9]. Recent data suggest UTI prevalence in LUTS-affected cats ranges from 2% to 19%, peaking at 40–45% in cats over 10 years [9]. 

Accurate diagnosis is essential to prevent misdiagnosis and inappropriate treatment [10]. FLUTD diagnosis relies on LUTS evaluation and requires bloodwork, urinalysis, abdominal ultrasound, and urine culture and sensitivity (UCS) testing to determine the underlying cause [9,11,12].

FLUTD significantly contributes to antimicrobial prescriptions in veterinary medicine, often leading to overuse in non-infectious cases such as FIC [10,13,14]. Historically, antimicrobial therapy was a primary treatment with broad-spectrum antibiotics and critically important antimicrobials (CIAs) [10,11]. However, growing awareness of infectious etiologies and antimicrobial resistance has shifted FLUTD management toward a multimodal approach, incorporating anti-inflammatory and nutraceutical therapies to reduce inflammation and support urinary health [15].

The aims of this observational study were as follows: (1) to collect the perspectives of Italian veterinarians regarding the clinical diagnostic approach and therapeutic management of FLUTD in non-obstructed cats and (2) to determine whether differences exist in diagnostic and therapeutic approaches across various work environments, focusing on antimicrobial prescriptions. We hypothesized that veterinarians across Italy commonly prescribe antimicrobials to cats with LUTS, and work environments differ in terms of diagnostic approach and choice of treatment for FLUTD.

## 2. Materials and Methods

### 2.1. Study Design

We conducted an observational cross-sectional study to gather information on the diagnostic approach and treatment of FLUTD in cats. An anonymous survey intended for veterinarians working in various regions of Italy was designed and distributed via a Google Form. The survey link was shared through forums and Provincial Professional Association mailing lists, accessible from 12 February to 10 March 2024. The questionnaire comprised 11 mandatory questions, including open-ended and multiple-choice formats (see Supplementary Materials—File S1).

The first three questions addressed the veterinarian’s work environment, including the region, province, and type of veterinary facility. Based on local legislation, the latter can be differenced into (a) small veterinary clinics (SC) that perform mainly primary care and only treat outpatients; (b) large veterinary clinics (LC) that perform both primary and secondary care on both in- and outpatients but do not have a 24 h emergency service; (c) veterinary hospitals (VH), mainly consisting of secondary and tertiary referral centers with 24 h emergency services.

The survey then focused on the diagnostic approach to FLUTD, offering options ranging from clinical signs alone to different combinations, including bloodwork, urinalysis, and abdominal ultrasound. The survey asked whether participants routinely performed urine culture and sensitivity (UCS) tests and, if not, to specify the reasons and challenges associated with this diagnostic step. 

Regarding treatment, the survey asked veterinarians to describe their therapeutic prescriptions, including antibiotics, anti-inflammatories, or supplements (e.g., D-mannose, probiotics). Particular attention was given to the class of antibiotics chosen for empirical treatment and the typical duration of therapy. Clinicians were then asked if they routinely performed UCS at the end of antimicrobial therapy. The last question addressed the use of antibiotics to manage urinary tract obstruction (UTO), aiming to estimate how frequently these patients receive empirical antimicrobial treatment.

### 2.2. Statistical Analysis

Descriptive analysis was performed, and results were reported as percentages. Post hoc analysis was conducted to compare different workplace settings using a chi-square test. The data were analyzed using commercially available software (GraphPad Prism 9). The significance was set at *p* < 0.05. 

## 3. Results

A total of 317 responses were collected. The distribution of answers between different regions of Italy is described in the Supplementary Material (Table S1). 

Two hundred respondents (63.1%) worked in an SC, 86/317 (27.1%) in an LC, and 31/317 (9.8%) in a VH. 

The diagnostic approach to FLUTD was guided by clinical signs and urinalysis in 151/317 (47.6%) cases, by clinical signs, bloodwork, urinalysis, and abdominal ultrasound in 121/317 (38.2%) cases, by clinical signs, bloodwork, and urinalysis in 29/317 (9.1%) cases, by clinical signs alone in 15/317 (4.7%) cases, and by clinical signs and bloodwork in 1/317 (0.3%) cases. Urinalysis was performed by 95% of the respondents. There was no significant difference between different workplaces (*p* = 0.104).

**Figure 1:**
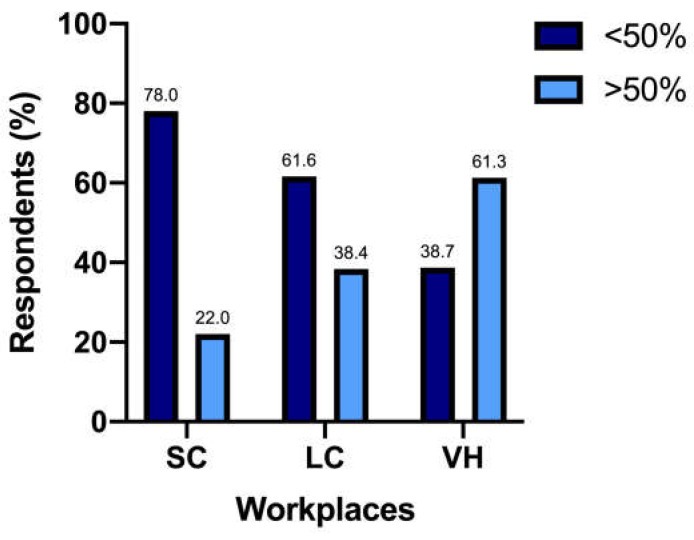
Responders use of UCS in more than 50% of patients in different work settings—SC, small clinics; LC, large clinics; VH, veterinary hospitals.

The UCS at the diagnosis was performed by 147/317 (46.4%) respondents in less than 25% of patients, by 74/317 (23.3%) in 25–50% of patients, by 46/317 (14.5%) in 50–75% of patients, and by 50/317 (15.8%) in more than 75% of patients. The percentage of respondents who performed UCS at diagnosis in more than 50% of cats differed significantly among SC (n = 44/200 [22%]), LC (n = 33/86 [38.4%]), and VH (n = 19/31 [61.3%]) (*p* < 0.0001) (Figure 1). 

The main reasons for not performing UCS included ‘owner’s financial constraints’ (n = 211/317 [66.6%]), ‘difficulties in urine collection’ (n = 87/317 [27.4%]), ‘not necessary’ (n = 60/317 [18.9%]), ‘delayed results’ (n = 57/317 [18%]), and ‘difficulties with shipping to an external laboratory’ (n = 15/317 [4.7%]). 

First-line treatment protocols prescribed by respondents are reported in Table 1.

Overall, antibiotics, anti-inflammatories, and supplements were included in the first-line treatment protocol by 109/317 (34.4%), 297/317 (93.7%), and 269/317 (84.8%) respondents, respectively. The empirically prescribed antibiotic classes are described in Table 2. 

**Figure 2:**
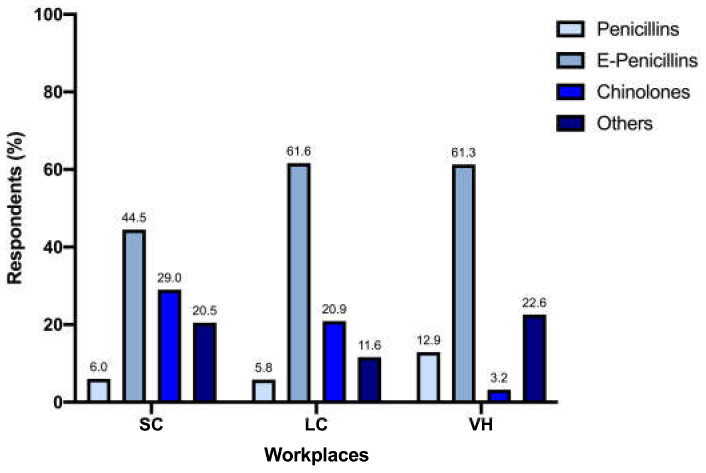
Prescription of enhanced penicillins, quinolones, and penicillins between workplaces.

**Figure 3:**
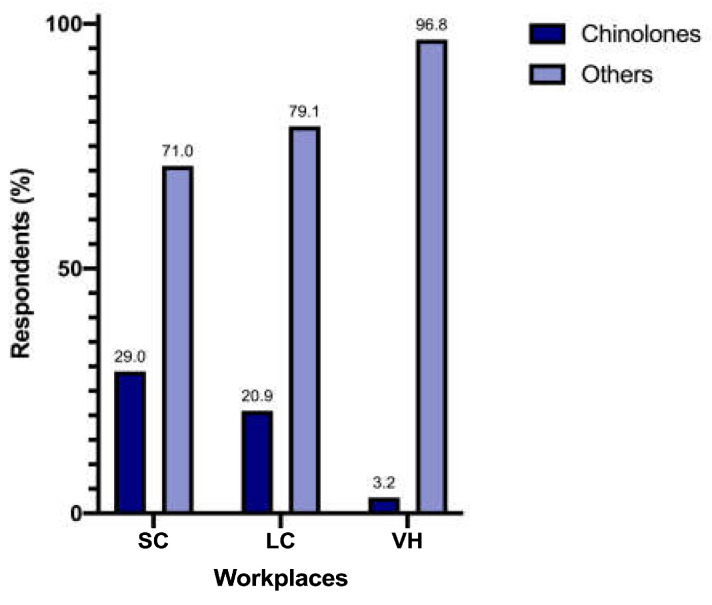
Prescription of quinolones between workplaces.

Prescription of enhanced penicillins, quinolones, and penicillins differed significantly between SC (n = 89/200 [44.5%], n = 58/200 [29%] and n = 12/200 [6%], respectively), LC (n = 53/86 [61.6%], n = 18/86 [20.9%] and n = 5/86 [5.8%], respectively), and VH (n = 19/31 [61.3%], n = 1/31 [3.2%] and n = 4/31 [12.9%], respectively) (*p* = 0.009) (Figure 2). When analyzed separately, the prescription of quinolones differed significantly between different workplaces (*p* = 0.005) (Figure 3). 

Regarding the duration of antibiotic therapy, 138/317 (43.5%) respondents indicated 7 days, 133/317 (42%) indicated 10–14 days, 32/317 (10.1%) indicated ≤ 5 days, and 14/317 (4.4%) indicated > 14 days. 

**Figure 4:**
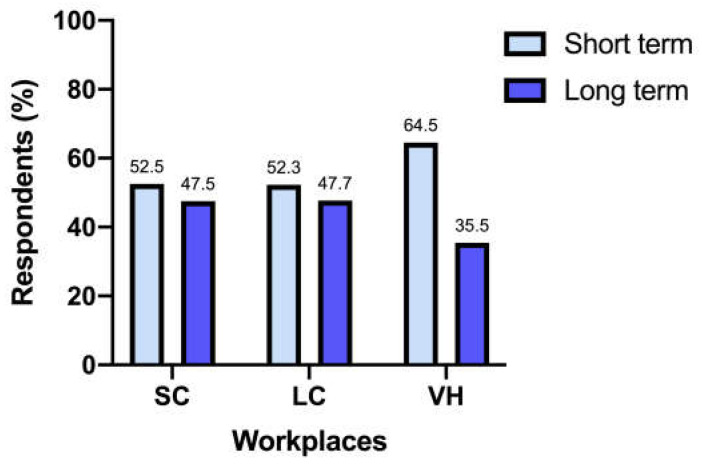
Length of antibiotic treatment between workplaces.

When grouped as short-term antibiotic treatment (7 days or less) and long-term antibiotic treatment (10 days or more), different workplaces did not differ significantly (*p* = 0.44) (Figure 4). 

**Figure 5:**
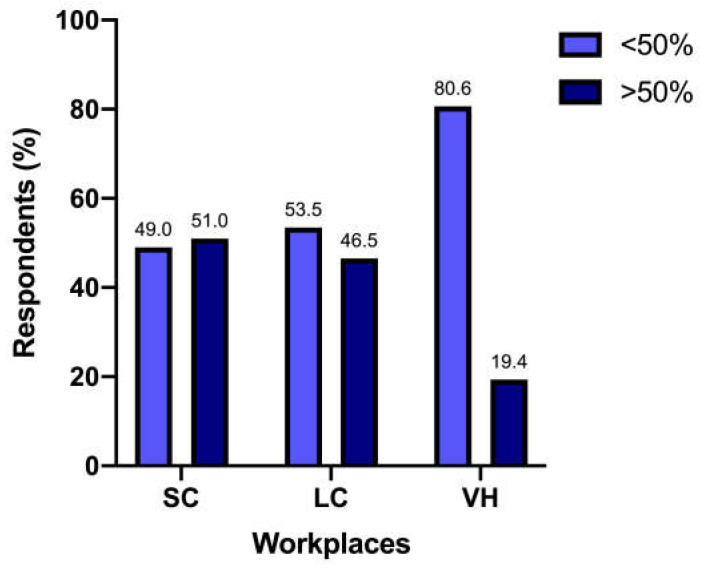
Antimicrobial treatment is prescribed for cats with UTO between different workplaces.

Antibiotics were prescribed for the management of cats with UTO by 98/317 (30.9%) respondents for less than 25% of patients, by 90/317 (28.4%) for more than 75% of patients, by 71/317 (22.4%) for 25–50% of patients, and by 58/317 (18.3%) for 50–75% of patients. When compared between different workplaces, the percentage of antimicrobials prescribed in cats with UTO differed significantly (*p* = 0.004) (Figure 5). 

## 4. Discussion

This Italian survey provides insights into FLUTD from the perspective of clinicians, focusing on diagnostic approach and treatment strategies. Once LUTS are identified, the diagnostic approach used by respondents to identify their etiology generally falls into two categories: slightly less than 50% rely primarily on clinical signs and urinalysis, while others employ additional methods such as bloodwork and abdominal ultrasound.

While obtaining a clinical diagnosis of FLUTD is simple, determining its underlying causes can be more complex. Comprehensive medical history, identification of behavioral criticalities, assessment of inflammation, and detection of possible uroliths via urinary ultrasound are critical steps that should not be overlooked [16]. The prevalence of bladder uroliths in cats with LUTS ranges from 12 to 23% [4,17,18,19,20]. Despite its value, ultrasound is used by only 38.2% (121/317) of respondents in their diagnostic workflow, which may occasionally lead to missed urolith detection and less tailored therapeutic management. While abdominal ultrasound may not always be immediately necessary at the first presentation, its inclusion becomes particularly important in cases of recurrent or persistent LUTS to support an accurate and timely diagnosis.

Urinalysis is considered a fundamental tool, with 95% of the respondents utilizing this exam. There was no significant difference between different workplaces. This result was unexpected considering the challenges associated with obtaining appropriate urine samples in cats; it may also reflect respondents’ inclination to report what they perceived as the “correct” answer rather than their actual clinical practices. Nonetheless, urinalysis is cheap and provides rapid results, and urine samples can often be collected by owners at home, reducing stress for the cat, which may contribute to higher reported utilization rates than expected.

Performing urinalysis is particularly crucial when assessing patients comprehensively, especially considering the presence of comorbidities that may influence urinalysis results and previous medical history. Recent studies suggest a higher prevalence of bacteriuria in feline symptomatic patients, ranging from 12% to 40%, differing from the previous data available [8]. Usually, these cats were older, increasing the possibility of comorbidities affecting their immune competence, such as metabolic disorders or endocrinopathies [8]. Predisposing comorbidity has been identified in 75–87% of cats with a urinary tract infection or subclinical bacteriuria [9,21]. The previous medical history of these feline patients and possible procedures performed previously must be considered, such as urethral catheterizations, that can raise the risk of bacterial colonization of the urinary tract [3]. 

The International Society for Companion Animal Infectious Diseases (ISCAID) guidelines outline evidence-based recommendations for the diagnosis and management of bacterial urinary tract infections in dogs and cats, aiming to mitigate antibiotic resistance [10]. Based on the ISCAID guidelines, the diagnosis of bacterial cystitis in cats with FLUTD should be based on positive urine culture due to the low prevalence of bacterial etiology, reported to be as low as 2% in young cats [2,5,8,9,10]. In our study, only 30.3% of respondents routinely (i.e., in more than 50% of cases) included UCS in their diagnostic protocols, mainly due to financial constraints and challenges in collecting an appropriate urine sample. Given financial constraints, owners may hesitate to invest a significant amount of money in diagnostic tests for infectious causes, especially when their prevalence is low. Additionally, obtaining a suitable urine sample of UCS can be challenging, since in FLUTD, the feline bladder is often empty due to extreme discomfort and pollakiuria. Given the high rate of urinalysis reported in our questionnaire, it is possible that owners find it easier to collect urine samples in a home environment. However, such samples are not suitable for UCS, as they may be contaminated and may not meet the sterility requirements necessary for accurate bacterial culture.

It has also to be noted that this percentage of respondents mostly worked in SC, with fewer coming from LC. The 61.3% of respondents working in VH, instead, relied on UCS in more than 50% of feline patients; the explanation for these numbers can probably be found with a greater awareness of guidelines in these different workplaces, along with the reasonable probability of more complex cases in VH, leading to a thorough medical approach [22].

Interestingly, 18.9% of responders did not consider UCS necessary for FLUTD evaluation. While this may align with the low prevalence of bacterial cystitis [2,5,8,9], it contrasts with the frequency of antimicrobial prescriptions reported for FLUTD in our questionnaire, with 34.4% using empirical antibiotics. In particular, the most common empirical choices were enhanced penicillins, quinolones, and penicillins. While part of these results may be in line with the ISCAID recommendations of using amoxicillin and amoxicillin-clavulanic acid (if not available alone) as the first-line empirical treatment for suspected bacterial sporadic cystitis [10], the ISCAID guidelines themselves do not suggest the empirical use of antimicrobials in cats with FLUTD, unless concurrent pyelonephritis is found. Moreover, the rate of prescription of quinolones is worrisome, considering they are not recommended as a first-choice treatment and their use should be based on UCS results and limited to situations where other molecules are unlikely to be effective (e.g., upper urinary tract infections, infections sustained by bacteria resistant to first-line antimicrobials) [10]. The once-daily administration is the most likely explanation for the overall use of this antimicrobial class, which is a convenient feature for feline owners and is consistent with the data available in the literature [10,14,22]. The same rationale can be hypothesized when third-generation cephalosporins are prescribed; in all cases, clinicians referred to cefovecin, distributed in long-acting injectable form. However, according to Italian legislation, these antimicrobials should only be prescribed based on the results of an antibiogram; empirical prescription should be avoided, considering the high risk of induced antimicrobial resistance linked to inappropriate use.

Unexpectedly, we have not found significant differences in the total amount of antimicrobial prescription between different workplaces; this, perhaps, can be linked to the different number of responders for each type of structure, creating an uneven comparison and leaving room for further analysis on this fundamental aspect. In contrast, differences in the choice of antimicrobial classes were noticeable, especially for quinolones. Veterinarians working in VH showed the lowest prescription rate: the reasons for difference were not investigated in this survey but could reflect a higher level of attention to antimicrobial stewardship and public health, as the recent literature recommends [23].

Weese et al. [22], in 2022, highlighted differences in antimicrobial prescription durations across workplaces. However, our survey did not find the same variations, with an overall length of therapy of 7 days in 43.5% of responders and 10 to 14 days in 42% of responders, regardless of the work environment. Cats suspected of having upper urinary tract infections might require longer antimicrobial courses; however, these cases should be correlated with evidence of kidney involvement, and UCS findings should guide the prescriptions. Notably, shorter antimicrobial courses are suggested by the 2019 ISCAID guidelines, highlighting the need for further alignment within the veterinary community [10].

When asked about the management of UTO, 28.4% of veterinarians reported prescribing antimicrobial therapy in more than 75% of cases. While studies consistently show a low prevalence of bacteriuria in cats with UTO, antimicrobial use may stem from efforts to prevent catheter-associated urinary tract infections (CA-UTIs) [3,24]. However, evidence suggests that this strategy does not reduce infection risk and can lead to pathogen selection and potentially antimicrobial resistance [3]. Consequently, the use of antibiotics for UTO management is not currently recommended and represents an area for improvement in clinical practice [3,9].

Comparing workplaces, we found a significant difference when looking for antibiotic prescriptions in more than 50% of patients who presented with UTO. This result further highlights the different attention to antimicrobial stewardship in different workplaces. Once again, the owner’s expectations and various workloads between SC, LC, and VH may influence this result and warrant further investigation to better understand the drivers behind these choices.

Due to its nature, this study has some limitations. Firstly, despite the anonymity of the survey, respondents may have been influenced to choose answers they perceived as ‘correct’, introducing bias and potentially skewing our assessment of actual clinical practices among Italian veterinarians. Second, we used the term “feline cystitis” in the questionnaire, referring to cats with FLUTD and LUTS. In Italy, the term “cystitis” is commonly used in a colloquial sense to refer to cats with LUTS, often encompassing various conditions associated with FLUTD. However, this lack of precision in terminology might have led to misinterpretations, potentially impacting the reliability of responses.

At the same time, we have not openly asked to limit the consideration to non-obstructive FLUTD, giving attention to UTO only regarding the prescription of antimicrobials in this subset of patients. Third, the choices for therapeutic strategies did not comprehend dietary choices and environmental management, but only drug choices and supplements; this should not lead to underestimating important areas of the multi-modal approach to FLUTD.

Finally, workplace comparisons were constrained by unequal respondent pools across SC, LC, and VH, necessitating cautious interpretation of statistical differences.

## 5. Conclusions

This study provides valuable insights into the diagnostic approach to FLUTD and the use of antibiotics among Italian veterinarians, highlighting adherence to and deviations from established guidelines such as those proposed by the ISCAID. Despite the high prevalence of idiopathic cystitis, which by definition has no known cause and is therefore not considered a bacterial condition, our results reveal a significant reliance on antimicrobial therapies, often administered without confirmatory UCS testing. The same problem is seen in the management of feline UTO. Encouragingly, larger facilities such as VH demonstrate good compliance with UCS-based diagnostics and antimicrobial stewardship. However, discrepancies persist in the selection and duration of antimicrobial therapies, with quinolones as an empirical treatment for FLUTD being a notable concern. These results underline the need for enhanced education on antimicrobial stewardship and adherence to evidence-based practices to minimize the risk of antimicrobial resistance.

## Figures and Tables

**Table 1:** Treatment choices based on 317 responses.

Treatments	Percentage of Respondents (n)
Anti-inflammatory and supplements ^1^	58.4% (185)
Antibiotics, anti-inflammatories, and supplements	20.8% (66)
Antibiotic and anti-inflammatory	9.8% (31)
Anti-inflammatory	4.7% (15)
Antibiotics and supplements	3.2% (10)
Supplements	2.5% (8)
Antibiotics	0.6% (2)

^1^ Supplements are considered dietary or nutraceutical products formulated to complement the diet with specific nutrients or bioactive compounds beneficial for supporting the health and functionality of the urinary tract. Examples used in the questionnaire are D-mannose and probiotics.

**Table 2:** Empirically prescribed antibiotic classes based on 317 responses (open answer).

Antibiotic Classes	Percentage of Responders (n)
Enhanced penicillins	51.7% (169)
Quinolones	25.4% (83)
Penicillins	6.4% (21)
First-generation cephalosporins	3.1% (10)
Third-generation cephalosporins	3.1% (10)
No empirical prescription	10.4% (34)

## Data Availability

Raw data are available on request.

## Supplementary Materials

The following supporting information can be downloaded at https://www.mdpi.com/article/10.3390/vetsci12040313/s1, File S1: Questionnaire distributed to veterinarians. Table S1: Number of responders from each region of Italy.
