# Supplementary material for: An Observational Study on the Diagnosis and Antibiotics Prescription in Cats with Lower Urinary Tract Disease by Veterinarians in Italy
Source: Vet Sci. 2025 Mar 30;12(4):313. doi: 10.3390/vetsci12040313 (PMC12031354; doi:10.3390/vetsci12040313)
Supplement: Supplementary file 1 [file vetsci-12-00313-s001.zip › File S1.pdf]

File S1

1. In which region of Italy do you work?
    - Choice from drop-down menu

2. In which province do you work?
    - Choice from drop-down menu

3. In what type of workplace do you work?
    - Small veterinary clinic
    - Large veterinary clinic
    - Veterinary hospital

4. How do you diagnose feline cystitis?
    - Clinical signs
    - Clinical signs + bloodwork
    - Clinical signs + urinalysis
    - Clinical signs + bloodwork + urinalysis
    - Clinical signs + bloodwork + urinalysis + abdominal ultrasound

5. Do you perform urine culture and sensitivity?
    - In more than 75% of cases
    - In 50–75% of cases
    - In 25–50% of cases
    - In less than 25% of cases

6. What are the most common reasons for not performing urine culture and sensitivity?
    - Owner's financial constraints
    - Difficulty in urine collection
    - Difficulty with shipping to an external laboratory
    - Delayed results
    - Not necessary

7. How do you typically treat feline cystitis?
    - Antibiotic
    - Anti-inflammatory
    - Antibiotic + anti-inflammatory
    - Complementary therapies (e.g., D-mannose/probiotics)
    - Antibiotic + complementary therapies (e.g., D-mannose/probiotics)
    - Anti-inflammatory + complementary therapies (e.g., D-mannose/probiotics)
    - Antibiotic + anti-inflammatory + complementary therapies (e.g., D-mannose/probiotics)

8. Which antibiotic do you empirically prescribe – while awaiting or in the absence of urine culture and sensitivity results?
    - Open answer

9. What is the average duration of antibiotic therapy you prescribe?
    - ≤ 5 days
    - 7 days
    - 10–14 days
    - 14 days

10. Do you perform urine culture and sensitivity at the end of antibiotic therapy?
    - In more than 75% of cases
    - In 50–75% of cases
    - In 25–50% of cases
    - In less than 25% of cases

11. Do you prescribe antibiotics for cats with urethral obstrusction? How often do you initiate antibiotic therapy during the management of an obstructed cat
    - In more than 75% of cases
    - In 50–75% of cases
    - In 25–50% of cases

- In less than 25% of cases
