# Supplementary material for: An Observational Study on the Diagnosis and Antibiotics Prescription in Cats with Lower Urinary Tract Disease by Veterinarians in Italy
Source: Vet Sci. 2025 Mar 30;12(4):313. doi: 10.3390/vetsci12040313 (PMC12031354; doi:10.3390/vetsci12040313)
Supplement: Supplementary file 1 [file vetsci-12-00313-s001.zip › Table S1.pdf]

Table S1 – answers between different regions

**Region**	**Answers**
Emilia-Romagna	58
Lombardia	57
Veneto	52
Toscana	44
Abruzzo	13
Piemonte	13
Umbria	12
Lazio	11
Campania	10
Friuli-Venezia Giulia	9
Marche	8
Calabria	7
Trentino-Alto Adige	7
Liguria	6
Sicilia	4
Puglia	3
Molise	1
Sardegna	0
Valle d’Aosta	0

Total answers: 317
